# Mechanism Exploration of 3-Hinge Gyral Formation and Pattern Recognition

**DOI:** 10.1093/texcom/tgab044

**Published:** 2021-07-03

**Authors:** Mir Jalil Razavi, Tianming Liu, Xianqiao Wang

**Affiliations:** Department of Mechanical Engineering, Binghamton University, Binghamton, NY 13902, USA; Cortical Architecture Imaging and Discovery Lab, Department of Computer Science and Bioimaging Research Center, The University of Georgia, Athens, GA 30602, USA; School of Environmental, Civil, Agricultural, and Mechanical Engineering, College of Engineering, the University of Georgia, Athens, GA 30602, USA

**Keywords:** 3-hinge gyri, axon fiber density, cortical folding, differential growth

## Abstract

The 3-hinge gyral folding is the conjunction of gyrus crest lines from three different orientations. Previous studies have not explored the possible mechanisms of formation of such 3-hinge gyri, which are preserved across species in primate brains. We develop a biomechanical model to mimic the formation of 3-hinge patterns on a real brain and determine how special types of 3-hinge patterns form in certain areas of the model. Our computational and experimental imaging results show that most tertiary convolutions and exact locations of 3-hinge patterns after growth and folding are unpredictable, but they help explain the consistency of locations and patterns of certain 3-hinge patterns. Growing fibers within the white matter is posited as a determining factor to affect the location and shape of these 3-hinge patterns. Even if the growing fibers do not exert strong enough forces to guide gyrification directly, they still may seed a heterogeneous growth profile that leads to the formation of 3-hinge patterns in specific locations. A minor difference in initial morphology between two growing model brains can lead to distinct numbers and locations of 3-hinge patterns after folding.

## Introduction

Cortical convolution or cortical folding is a prominent characteristic of the mammalian cerebral cortex. There are several studies that have discussed that the study of cortical folding could help us to better understand the normal development of the human brain during the gestation period (Armstrong et al. 1993; Harris et al. 2004; Duchesnay et al. 2007; Wisco et al. 2007; Fischl et al. 2008; Geng et al. 2009; Hu et al. 2010; Xu et al. 2010; Zilles et al. 2013; Bayly et al. 2014). Abnormal cortical folding in the fetal stage leads to cognitive or physiological difficulties and problems, for example, epilepsy, intellectual disabilities and autism spectrum disorder (ASD), and schizophrenia (Kulynych et al. 1997; Levine and Barnes 1999; White et al. 2003; Harris et al. 2004; Tortori-Donati et al. 2005; Kapellou et al. 2006; Schaer et al. 2006; Van Essen et al. 2006; Nordahl et al. 2007; Wisco et al. 2007; Csernansky et al. 2008; Guerrini et al. 2008; Kennedy and Courchesne 2008; Pang et al. 2008; Raybaud and Widjaja 2011; Sun and Hevner 2014; Budday 2014a).

Cortical folding has multiple stages: primary, secondary, and tertiary, which all take several months to complete (Sun and Hevner 2014; Budday et al. 2015c). Primary folding has been studied very well, while secondary and tertiary folding mechanisms are still quite unclear, leading to a need for more investigations into the development of the developing complex morphology. Primary folding is notably preserved among individuals (Lohmann et al. 2008), while secondary and tertiary folding evolve after primary folding is completed and can vary widely across individuals (Gilles et al. 1983). It is believed that differential growth within the cortex is a possible stimulus for secondary and tertiary folding (Richman et al. 1975; Tallinen et al. 2016). According to the tangential differential growth hypothesis, the outer layers of brain show more rapid growth than the inner layers. This growth rate mismatch acts as the driving mechanism for brain structure instability and gyrification. The tangential differential growth hypothesis has been proven by recent experimental and computational analyses (Ronan et al. 2014; Razavi et al. 2015a, 2015b; Tallinen et al. 2016), although it is believed that other factors such as axon fiber position and growth also play a nonnegligible role in the regulation of morphology (Holland et al. 2015; Zhang et al. 2016, 2017).

**Figure 1:**
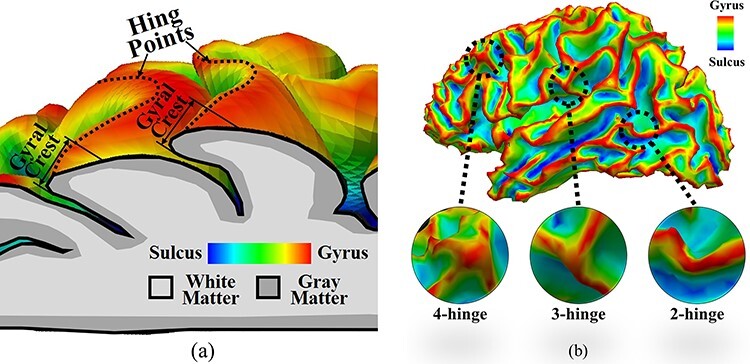
(*a*) An illustration to show the concepts of hinge points, hinge lines (dotted lines), and gyrus crest. (*b*) Example illustrations for 2-hinge, 3-hinge, and 4-hinge patterns.

Folding patterns of the human cerebral cortex show quite different morphologies between subjects in secondary and tertiary foldings (Van Essen et al. 1998; Fischl et al. 1999; Liu et al. 2004). Evidence also has shown that the folding patterns of the human cerebral cortex can predict its cytoarchitecture (Fischl et al. 2008). Therefore, understanding of the underlying mechanism and quantitative description of folding patterns are two important research goals. Before outlining the research subject, it is necessary to define some concepts, for example, “hinge point.” A hinge point is the point of minimum radius of curvature for a fold, and when these hinge points on a fold surface connect to each other, a hinge line is formed. As shown in Figure 1*a*, the hinge of a fold is the field of marked curvature adjacent to the hinge line (Li et al. 2010). Therefore, we can classify human gyral folding patterns into three classes according to their numbers of hinges: 2-hinge, 3-hinge, and 4-hinge gyri, Figure 1*b*. It has been shown in the literature, including our recent work (Li et al. 2010), that the formation of folding patterns on the cortex at the mesoscale and gyral-scale varies greatly among individuals. Therefore, the number of hinge lines connecting to a field can be used to describe the folding pattern of a hinge field on a gyrus (Yu et al. 2013). It is noteworthy that the 2-hinge structure is degenerated with the hinge line and that four gyral crests rarely meet to form 4-hinge gyri (Li et al. 2010). Thus, we will turn our attention to the most common identifiable structure, the 3-hinge pattern. In contrast to ordinary gyri, gyral hinges are of importance because they have the thickest cortices, the strongest long-range axonal connections and the most pronounced connective diversity, and the most aggregative functional profiles (Yu et al. 2013; Chen et al. 2014; Jiang et al. 2015, 2018; Li et al. 2017; Ge et al. 2018). Moreover, these gyral hinges behave more like cortical hubs in the corticocortical networks and compose a majority portion of the network’s “core” (Zhang et al. 2020). Quantitative characterization of gyral folding patterns via hinge numbers with cortical surfaces constructed from MRI data has been used to identify 6 common 3-hinge gyral folds that exhibit consistent anatomical locations across humans, chimpanzees, and macaques, as well as 2 unique 3-hinge patterns in macaques, 6 in chimpanzees, and 14 in humans (Li et al. 2017). It is not surprising that the number of 3-hinge patterns identified in the human brain is 2.5 and 7.8 times greater than the number found in chimpanzee and macaque brains, respectively. Therefore, the shape and number of 3-hinge patterns in a growing brain could be a new metric to characterize the folding of a primate brain. Interestingly, although there is a direct relationship between the number of 3-hinge patterns and brain size across these three species (human, chimpanzee, and macaque), the analysis of number of 3-hinge patterns in other species does not show a clear association between brain size and number of 3-hinge patterns (Li et al. 2017). In our previous work, we focused on the potential contribution of the dense growing axon fibers to the 3-hinges formation besides the differential growth in white and gray matters. However, it has not addressed the consistency of locations and patterns of certain 3-hinge patterns we have observed in cortical folding. Therefore, there is a critical need to develop a unified biomechanical principle to explain how the intrinsic relationships between cortical folding and structural connection patterns in brains lead to the formation of the observed highly convoluted 3-hinge patterns and to provide a novel diagnostic for neurological disorders useable during early brain development. As an example, by a comparative MRI study among control group and ASD group in their gyral hinge morphology, we observed that the identified difference in morphology and spatial distribution of 3-hinge patterns of ASD group is associated with the reported functional and cognitive differences (Huang et al. 2019). The study helps explain that the gyral hinges could be related to brain functions and used as a potential indicator for diagnostics.

In the present work, we test the hypothesis that the dense growing axon fiber pattern may regulate the patterns of the 3-hinges and help form specific types of 3-hinges and correlate the location, number, and shape of 3-hinges with the fiber patterns. We develop a biomechanical model to reveal underlying mechanism of the formation of 3-hinge gyral patterns. The geometrical and mechanical parameters are investigated to understand how these parameters control the number, location, and shapes of 3-hinge patterns. In order to do so, along with the biomechanical model, we develop an algorithm to automatically detect the number, location, and shapes of 3-hinge gyral patterns in *in-silico* models and in the reconstructed 3D images of real brain. Moreover, we try to elaborate the structural consistency and inconsistency concepts using mechanical simulation to explain the phenomenon where two individuals with globally distinct number and shape of 3-hinges patterns might show certain preserved 3-hinges patterns.

## Computational Models and Methods

### Constitutive Relationship and Governing Equation of a Growing Brain Model

Nonlinear finite element simulation accompanied by the theory of finite growth was developed to mimic growth and folding of a developing brain. By implementing the theory of multiplicative decomposition (Rodriguez et al. 1994), the deformation gradient $\boldsymbol{F}(\boldsymbol{X})$ and Jacobian $J$ are decomposed into an elastic element and a growth element. The elastic element describes pure deformation resulting from stresses, and the growth element indicates the addition of materials.

(1) \begin{equation*} \boldsymbol{F}=\boldsymbol{A}.\boldsymbol{G} \end{equation*}

(2) \begin{equation*} J=\det\ \left(\boldsymbol{F}\right)={J}^e{J}^g \end{equation*}

Here, $\boldsymbol{F}=\partial \boldsymbol{x}/\partial \boldsymbol{X}$. Points $\boldsymbol{X}$ from the undeformed configuration are mapped to their new positions $\boldsymbol{x}$ in the deformed configuration. Although both $\boldsymbol{A}$ and $\boldsymbol{G}$ tensors may be incompatible deformations, their multiplication, $\boldsymbol{F}$, should be a compatible deformation (Ben Amar and Goriely 2005). We assume that growth in the cortex is isotropic and defined by the growth ratio, $g$, in the growth tensor as:

(3) \begin{equation*} \boldsymbol{G}=g\boldsymbol{I} \end{equation*}

(4) \begin{equation*} {J}^g=\det\ \left(\boldsymbol{G}\right)={g}^3 \end{equation*}

Here, $g$ is a scalar. According to our previous works (Zhang et al. 2016; Ge et al. 2018), we model the material property of the brain with a hyperelastic material incorporating a strain energy function $W(\boldsymbol{A})$. We characterize the constitutive behavior through the following neo-Hookean free energy equation, parameterized exclusively in terms of the elastic tensor $\boldsymbol{A}$ and its Jacobian $J$.

(5) \begin{equation*} W=\frac{1}{2}\lambda{\ln}^2J+\frac{1}{2}\mu \left[\boldsymbol{A}:\boldsymbol{A}-3-2\ln J\right] \end{equation*}

Here, $\lambda$ and $\mu$ are Lamé constants. Following standard arguments of thermodynamics, the Piola stress $\boldsymbol{P}$ follows as energetically conjugate to the deformation gradient:

(6) \begin{equation*} \boldsymbol{P}=J\frac{\partial W}{\partial \boldsymbol{F}}\cdot{\boldsymbol{G}}^{-\mathrm{T}} \end{equation*}

In the absence of body forces, mechanical equilibrium imposes:

(7) \begin{equation*} \mathrm{Div}\ \boldsymbol{P}=0 \end{equation*}

For all parts of the model (white matter, gray matter, and axonal fibers), a similar shear modulus of 0.5 kPa was used (Budday et al. 2015c).

### Computational Model of a Growing Brain

**Figure 2:**
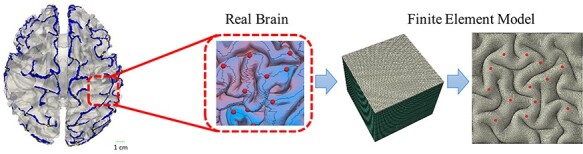
A piece of brain selected to construct finite element model.

We constructed a three-dimensional (3D) double-layer model as a small piece of the brain to explore the fundamental mechanism of cortical folding and 3-hinge pattern formation, as demonstrated in Figure 2. This model has previously been used to study consistent gyrus formation and also fiber density effect on 3-hinge formation (Ge et al. 2018). Since the main purpose of this study is to investigate mechanisms of 3-hinge pattern formation rather than the effect of the geometry of the model, a flat structure was selected despite the fact that curvature has a considerable effect on convolution patterning (Budday et al. 2015c). In the finite element model, a thin top layer represents the cortex (cortical plate). The bottom layer is the core, which is supposed to be a simple representation of the subplate, intermediate zone, and ventricular zone. In human brains, the cerebral cortex is a thin ($2\hbox{--} 4\ \mathrm{mm}$) layer (Bayly et al. 2014), in contrast to the core that has a much greater thickness of around $50\ \mathrm{mm}$ (Tallinen et al. 2014). The dimension of the model was selected based on experimental data gathered from small pieces of a brain. The dimension of the base model was $60\ \mathrm{mm}\times 60\ \mathrm{mm}\times 50\ \mathrm{mm}$ (not including cortical thickness), and the thickness of the cortex was variable across different models in order to trace the relationship between cortical thickness and the number of 3-hinge patterns. The thickness of the cortex and white matter before cortical folding was set to $1.5$ and $50\ \mathrm{mm}$, respectively. However, in the study of the effect of cortex’s thickness on the 3-hinge geometrical and mechanical features, the initial cortex’s thickness is set to vary from $1\ \mathrm{to}\ 2\ \mathrm{mm}$. Symmetric boundary conditions were applied on four sides of the model and the bottom surface of the core was fixed. The dimension of the model was large enough in comparison with the wavelength of folded patterns observed in experiments so as to prevent boundary effects. In a bilayer model with an isotropic growth for both cortex and subcortex without stress-dependent growth and no boundary confinement, only their relative growth ratio is a determinant factor for instability and folding. Also in a human premature brain, the volume of the cortical plate increases by fourfold in the first 2 months (22–30 GWs), while the subplate plus intermediate zone (SP + IZ) increases approximately by threefold (Scott et al. 2011). In our recent analytical–computational study, we have already shown that the growth ratio of 4/3 between two distinct layers is sufficient enough to trigger the structural instability (Razavi, Zhang, Li, et al. 2015a). This ratio is independent of the growth ratios’ absolute values. Without loss of generality, we set the cortex to grow gradually with no growth in the core (Tallinen et al. 2014; Zhang et al. 2016). The growth of the cortex was assumed to be linear in time:

(8) \begin{equation*} {\dot{g}}_{\mathrm{cortex}}={g}_{\mathrm{ct}} \end{equation*}

Here, ${g}_{\mathrm{ct}}$ is a cortex growth constant rate. Previous study has shown that brain growth and folding take place in a long period of time without immediate growth activation (Zöllner et al. 2012). Axonal fibers can elongate under the tensional axial forces as a soft slender structure, but their creep behavior and change in the cross-section by the time due to stress accumulation are not modeled in this study (Lamoureux et al. 2010). We believe that the assumption, namely decoupling the axonal fiber growth from stress state, does not have a considerable effect on the results of the study. Another point is worthwhile to mention that the behavior of axonal fibers inside the surrounding GS might differ from what we can see from the microneedle experiments (Dennerll et al. 1989). Experimental studies (Bray 1984; Dennerll et al. 1989; Lamoureux et al. 2010) have shown that axonal fibers under deformation show a towed growth response (Zöllner et al. 2012), chronic lengthening/shortening to maintain a desired level of axonal tension. However, because cortical folding completes in a long time scale, as the first-order approximation, a nonlinear hyperelastic material model without tension-driven growth has been adopted to study the behavior of the axonal fibers in this study similar to the other micromechanical modeling studies including axonal fibers (Pan et al. 2013; Ahmadian 2018, Hoursan et al. 2020; Yousefsani et al. 2018).

Growth in the model was simulated by thermal expansion (Razavi and Wang 2015; Razavi et al. 2015a). For further understanding of the analogy between the volumetric growth model and thermal stress model, please check reference (Cao et al. 2012a, 2012b). By adjusting the thermal expansion coefficient to the cortex and increasing temperature in dynamic steps, the cortex expands, destabilizes, and then starts to fold. Finite element models were constructed, meshed, and solved using the ABAQUS FE package, which is suitable for large deformation, nonlinear, and quasistatic problems. The mesh size was selected as small enough to make qualitative features of the model independent from mesh size. Material properties of the cortex and core were supposed to be same, since there is no substantial difference between gray matter and white matter material properties (Budday et al. 2015b; Tallinen et al. 2016).

A series of initial small perturbations were introduced into the models to check the effect of initial perturbations on 3-hinge patterns after convolution. Convolution patterns after instability are not guaranteed to be exactly symmetric although the initial model is symmetric (Tallinen et al. 2013; Tallinen and Biggins 2015). Applying a small initial perturbation (e.g., displacement perturbation, force perturbation, or mech defects) in mechanical models for triggering instability is a common method. There are a lot of studies that have applied small initial perturbations in their growth models for mimicking the biological or mechanical postperturbation behaviors (Cai et al. 2012; Zang et al. 2012; Cao, Jia, et al. 2012a; Bayly et al. 2013; Budday et al. 2014b; Wang and Zhao 2015; Budday, Kuhl, et al. 2015a). In our model brain, we apply a small initial perturbation as a trigger of instability after growth. In a real brain, this perturbation could stem from the variation of curvature or heterogeneous growth on distant sites of the brain. The amplitude of the checkerboard perturbation (${\epsilon}_z$) applied in the model brains is 1% of the thickness with a relation as follows:

(9) \begin{equation*} {\epsilon}_z={A}_0\left[\sin \left(\omega x\right)+\sin \left(\omega y\right)\right] \end{equation*}

where ${A}_0$is the amplitude and ω is the wavelength. The subscript “*z*” indicates the normal direction to the cortex surface. The ω in each 30 models is different, so every model has different initial perturbations.

There are two types of growths in axon fiber bundles: 1) growth due to mechanical tension and 2) intrinsic biological growth. For the first type of growth, fiber bundles emanate from the base of the model all the way to the interface of the cortex and white matter, which are assumed to be elastic and can grow due to mechanical stress, called the tension-based growth. This tension-based growth in axon fiber bundles depends on the mechanical stress state of cortical foldings and therefore dynamically varies with the stress state changes. With respect to biological intrinsic growth, we assumed that axon fiber grows along the fiber axial direction and assigns a thermal expansion to mimic it ( Cao, Jia, et al. 2012a). In this study, we set the biological intrinsic growth to be a constant growth. Growth in axonal fibers is defined as:

(10) \begin{equation*} {\dot{g}}_{\mathrm{axon}}={g}_{\mathrm{af}} \end{equation*}

where ${g}_{\mathrm{af}}$ is an axonal fiber growth constant rate. Therefore, the growth tensor for the axonal fiber is:

(11) \begin{equation*} \boldsymbol{G}=\left({g}_{\mathrm{axon}}-1\right)\boldsymbol{z}\otimes \boldsymbol{z}+\boldsymbol{I} \end{equation*}

In the models, ***z*** shows a unit vector aligned with the axial direction. Increasing the temperature by time causes only the axonal fibers to grow in axial direction.

However, with combined tension-based growth and the intrinsic growth, the resultant growth of axon fiber bundles varies across the entire cortex and along the entire process of cortical folding. As demonstrated in our prior study (Ge et al. 2018), the axon fiber density concentration has been observed to be highest at the hinge point of 3-hinge patterns and decays gradually along the hinge spoke. Therefore, in our model brain, we assumed that the hinge junction has the highest axial intrinsic growth with its growth rate being equal to the growth rate of the cortex. ${g}_{\mathrm{axon}}$is the maximum growth ratio at the 3-hinge junctions accompanied by growth ratios along the hinge lines decaying proportionally from ${g}_{\mathrm{axon}}$ to 0. The growth in the fiber bundles is not isotropic. The hinge lines that include fibers grow only in the axial direction (axial direction is normal to the surface of the cortex). In this study, we also assume that the scaler value of ${g}_{\mathrm{af}}$ of axonal fibers is equal to ${g}_{\mathrm{ct}}$.

### Brain Imaging Data and Preprocessing

The Q3 release of Human Connectome Project (https://www.humanconnectome.org) data was used in this study. All HCP subjects were scanned on a customized Siemens 3 T “Connectome Skyra.” As we focused on cortical morphology, the white matter surfaces reconstructed from structural MR scans of 868 healthy subjects were of our major interest. Important structural session imaging parameters are listed in Table 1. The white matter surfaces have been produced via the version 3 preprocessing pipelines. Diffusion MRI data were also used to exhibit the distribution of axonal fibers shown. A full DTI session includes 6 runs, representing 3 different gradient tables. Each gradient table includes approximately 90 diffusion weighting directions plus 6 *b* = 0 acquisitions interspersed throughout each run. It consists of three shells of *b* = 1000, 2000, and 3000 s/mm^2^ interspersed with an approximately equal number of acquisitions on each shell.

**Table 1:** Structural session imaging parameters in HCP 868 dataset

Type	Description	TR (ms)	TE (ms)	Flip angle	FOV (mm)	Voxel size	Acquisition time (min:s)
T1w	3D MPRAGE	2400	2.14	8 deg	224 × 224	0.7 mm isotropic	7:40
T2w	3D T2-SPACE	3200	565	Variable	224 × 224	0.7 mm isotropic	8:24

The white matter surfaces reconstructed from structural MR scans were provided by HCP dataset by following the structural protocols and data preprocessing pipelines in WU-Minn HCP 900 Subjects Data Release. To produce the fiber density profile from diffusion MRI, we firstly performed skull removal, motion correction, eddy current correction via FSL (Jenkinson et al. 2012; Andersson and Sotiropoulos 2016). The model-free generalized Q-sampling imaging (GQI) method (Yeh et al. 2010) in DSI Studio was then used to estimate the diffusing orientations. Next, the deterministic streamline tracking algorithm in DSI Studio (Yeh et al. 2013) was used to reconstruct 4 × 10^4^ fiber tracts for each subject using the default fiber tracking parameters (max turning angle = 60^o^, streamline length between 30 and 300 mm, step length = 1 mm, and quantitative anisotropy threshold = 0.2). To estimate the fiber density map, we also reconstructed the white matter surface from fractional anisotropic (FA) map of the diffusion MRI. Specifically, FA map was derived from diffusion MRI via FSL-FDT (Jenkinson et al. 2012), first. The FA value quantifies the extent to which water molecules diffuse within a voxel (Beaulieu and Allen 1994; Beaulieu 2002). Next, tissue segmentation (white matter, gray matter, and cerebrospinal fluid, CSF for short) was performed on FA map via FSL-FAST (Zhang et al. 2001). Finally, based on the segmentation results, the white matter surface, the border between the white matters and gray matters, was reconstructed via our home-made surface reconstruction toolkit (Liu et al. 2008). Fiber density was defined as the number of streamline fibers that penetrate a unit surface area.

### Imaging Data Analysis Methods

**Figure 3:**
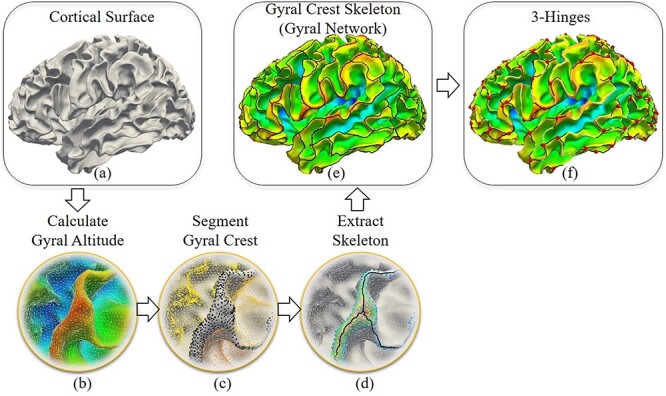
Pipelines of the extraction of the gyral skeleton and 3-hinge patterns. (*a*) Cortical surface of the white matter/gray matter boundaries. (*b*) and (*d*) Gyral crest segmentation and skeletonization on an enlarged region. (*b*) Presentation of gyral altitudes. Red regions have positive altitude values, while blue regions have negative altitude values. (*c*) The cortical surface is segmented into gyral crests (black dots). (*d*) Gyral crest skeleton extraction (black curves). (*e*) The gyral crest skeleton on a brain. (*f*) The presentation of 3-hinge patterns’ centers (red dots).

In general, 3-hinge data are based on the result of gyral net extraction *via* our recently developed automatic pipeline (Chen et al. 2017). The aim of gyral net extraction is to separate gyral crests from other cortical regions and then skeletonize them to present them as a gyral network. The 3-hinge patterns are defined as the joints of such network for which the degree of connection is equal to 3. Briefly, the pipeline consists of two major steps: 1) Gyral crest segmentation: We defined the “midsurface” as a line that separates gyri and sulci. This “midsurface” is chosen so that the mean of the displacements of all surface vertices from their original locations is zero. Gyral altitude for a vertex is defined as the movement from its original location to the “midsurface” in the surface normal direction, Figure 3*b*. Based on these definitions, the watershed algorithm (Bertrand 2005) is adopted to segment the gyral crest (regions over an altitude level, black dots in Fig. 3*c*). More details and effects of the watershed algorithm can be found in our previous works (Chen et al. 2017). 2) Gyral skeleton extraction: This step is to skeletonize gyral crests, as seen in Figure 3*d*,*e*. Gyral skeletons are defined as the crest curves located in the central parts of gyral crest. First, a distance transformation algorithm was conducted on the segmented gyral crests to highlight their central regions. Then, a tree marching algorithm was adopted to successively connect the vertices to form multiple tree-shape graphs. After the redundant branches were pruned, the major branches were left and taken as the skeleton of the gyral crests.

This skeleton can be taken as a gyral network. We defined vertices on this network with degrees more than 2 as gyral joints, red dots in Figure 3*f*. Gyral joints with degrees equal to 3 were defined as 3-hinge patterns and are the major interest of this work.

### Detection of Location and Shape of 3-Hinge Patterns

**Figure 4:**
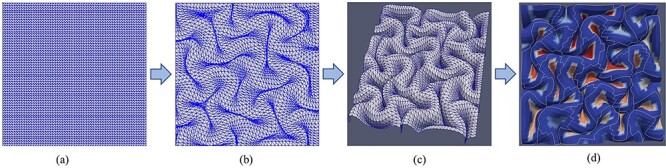
Process of 3-hinge patterns’ detection. (*a*) Initial state of the meshed area created by triangular mesh. (*b*) The deformed coordinates of the cortical surface in ABAQUS are reconstructed in MATLAB. (*c*) The constructed surface is visualized by Paraview software. (*d*) A developed algorithm detects path lines and center points of 3-hinge patterns. Blue to red shows a transition from convex curvature to concave curvature.

Several FE models with different initial perturbations were run to find geometrical specifications of 3-hinges in a developing brain. Node coordinates of FE models were extracted and fed as input to a MATLAB code to construct a surface of the convoluted model. Then constructed surfaces were fed to a developed algorithm (Chen et al. 2017) to detect numbers and shapes of 3-hinge patterns. Figure 4 schematically shows how the numbers and the shapes of 3-hinge patterns are extracted from the FE models.

### Feature Extraction and the Shape Classification of 3-Hinges

Pipeline for the feature extraction and the shape classification of 3-hinges has been discussed in detail in our previous work (Zhang et al. 2018). For details, the rationales and parameter settings are referred to our work by Zhang et al. (2018).

### Statistical Analysis

Imaging data results are presented as arithmetic mean averaged over 868 human subjects with no outliers. Least mean square curve fitting method was used to produce the trend lines that fit the data points. Imaging data were analyzed using FreeSurfer and MATLAB (R2015a). Computational data results are presented as arithmetic mean. Least mean square curve fitting method was used to produce the trend lines that fit the data points. ABAQUS toolkit was used to execute the computational experiments.

## Results and Discussions

### Formation of 3-Hinge Patterns in the Human Brain

**Figure 5:**
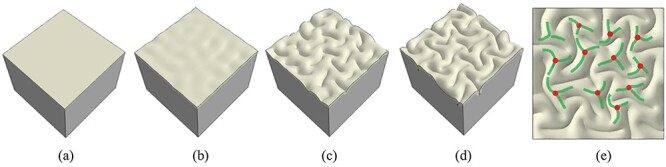
Morphological evolution of a growing brain model. (*a*) Initial perturbation before simulation. (*b*) Instability initiation. (*c*) Folding after instability. (*d*) Formation of convoluted patterns and 3-hinge patterns. (*e*) Top view of the highly convoluted cortex. Some 3-hinge patterns are detected on the surface. Red points show centers of 3-hinge patterns and green curves show their patterns.

In order to single out the mechanism of formation of 3-hinge patterns in a human brain, we first performed a series of finite element (FE) simulations with homogeneous growth in the cortex. Therefore, a small part of the growing brain is mimicked by a flat bilayer plate model (see Computational Model of a Growing Brain section). The reason for considering a small flat patch is that the focus of study is on the underlying mechanism of formation of 3-hinge patterns rather than the effect of brain irregular geometry. Using simple geometry could give basic knowledge regarding growth, folding, and formation of 3-hinges in a brain. The thickness of the cortex in the initial state was considered to be$1.5\ \mathrm{mm}$. This number was found based on trial and error so that the average thickness of the cortex after convolution is close to $3.5\ \mathrm{mm}$ (the average cortical thickness of a mature human brain) (Bayly et al. 2014). Figure 5 shows a typical evolution process of the growing brain model and the 3-hinge pattern formation: 1) The cortex gradually grows under a small applied initial perturbation (check Methods Section for details of initial perturbation); 2) instability is initiated on the cortex after a critical growth ratio due to the considerable induced compressive stresses; 3) growth within the cortex after instability leads to folds, producing gyri and sulci; 4) with increasing time and growth, folds become more convoluted and self-contacting folds form 3-hinge patterns. Red points in Figure 5*e* show the locations of 3-hinge centers and the green curves denote hinge lines.

**Figure 6:**
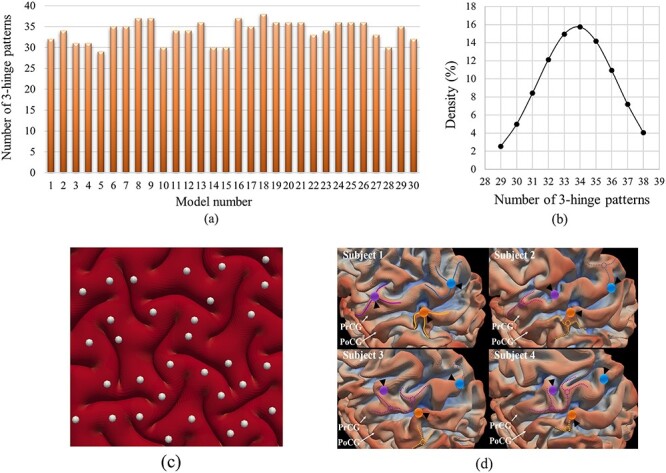
(*a*) Dependency of numbers of 3-hinge patterns on small initial perturbation. (*b*) Normal distribution of the number of 3-hinge patterns. (*c*) An example case to show how the developed algorithm automatically detects the locations of 3-hinge patterns. (*d*) Variation in location of 3-hinge patterns on the precentral gyrus (PrCG) and postcentral gyrus (PoCG) for four randomly selected subjects. The cortical surfaces of four subjects have been aligned to the gray-ordinate standard system by the HCP preprocessing pipeline (corresponding vertices are of the same ID). We manually identified three 3-hinge patterns on subject #1. The hinge centers are represented by color bubbles. The hinge spokes are represented by solid curves. The anatomical corresponding vertices on subjects #2-#4 via HCP preprocessing pipeline are highlighted by the corresponding color bubbles and black arrows. We also manually identified the corresponding 3-hinges on subjects #2-#4, the centers and spokes of which are represented by dashed circles and curves and color-coded by the correspondences. This figure has been reused with permission from Ref. (Zhang et al. 2018).

Thirty models with different small initial perturbations were run while all other parameters were kept the same. After computation and postprocessing of all models, the number and location of 3-hinge patterns in every model were calculated by the developed algorithm (see Methods section). Results are shown in Figure 6*a* which illustrates that with different initial perturbations, different numbers of 3-hinge patterns form on the cortex. The number of 3-hinges patterns ranged from 29 to 38 and the average number is around 34. Figure 6*b* shows the normal distribution of the number of 3-hinge patterns. The image in Figure 6*c* shows an example case of how our automatic algorithm has detected the locations of the 3-hinge patterns. Results indicate that in all models, the size and thickness of gyri in the hinge lines in 3-hinge patterns are similar to each other, but locations and shapes can be quite different. The similar variation of locations of 3-hinge patterns can be observed in different individual brains. As an example, Figure 6*d* shows how the locations of major 3-hinge patterns are different in four randomly selected subjects. These results may explain why 3-hinge patterns are notably different across individuals even when they are quite similar in the size and thickness of gyri in the hinge lines. In other words, a very small difference in the initial smooth brain can lead to the formation of considerably different 3-hinge patterns after the development.

**Figure 7:**
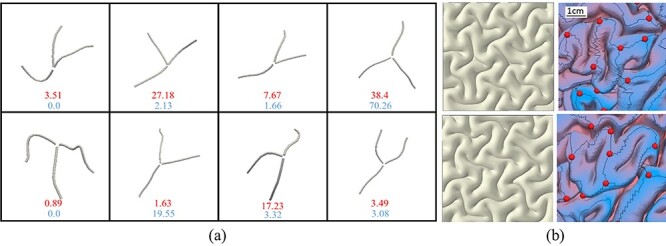
(*a*) Dominant shapes of 3-hinge patterns in real brains and FE models. The top numbers (red color) and bottom numbers (blue color) are the percentages found in real brains and FE models, respectively. (*b*) Comparison of convolution patterns between our FE models (left) and experimental images of real brains (right). In the experimental images, gray matter has been removed for easier analysis of gyral crests.

In addition to the number of 3-hinges, using the FE data, we have also extracted the dominant 3-hinge patterns in order to create a comparison with those observed in the brain imaging data. For the experimental analysis, the dominant 3-hinge patterns and their relative occurrence percentages were extracted from 68 brain subjects with 7498 detected 3-hinge patterns. For the computational analysis, from the FE models, 844 3-hinge patterns were detected and extracted. Figure 7*a* shows eight dominant shapes of 3-hinge gyral patterns identified in the human brain and FE models. The top numbers (red) and bottom numbers (blue) are the percentages found in real brains and FE models, respectively. This comparison indicates that “Y”-shaped 3-hinge patterns are the most favorable patterns found in real brains as well as in FE models, although overall the real brain demonstrates more variety in terms of 3-hinge patterns’ shapes. These results show that our biomechanical model, at least partially, can mimic the growth of a brain and capture complex convoluted 3-hinge patterns. With a careful look at Figure 7*a*, it can be observed that real brains have certain 3-hinge pattern shapes that our FE models do not capture, although the observed percentage of such shapes is low even in data from real brains. Therefore, there should be other factors that regulate 3-hinge gyral patterns in real brains, which are not yet included in our FE models. We will discuss these factors in the following sections. Figure 7*b* qualitatively compares convolution patterns between randomly selected FE models and experimental images. The FE models have different initial perturbations before growth, but all other parameters are the same. The experimental images are extracted from the frontal lobes of real brains. Figure 7*b* indicates that FE models with small initial perturbations develop different convolution patterns. From the mechanical view, as folding process is a dynamic process, final patterns are highly dependent on the initial states and imperfections. Initial small perturbations in the FE model may be analogous to differences in curvature for real brains. This means that different parts of a brain, without considering any other factors and just by attention to their location and curvature, are able to develop different folding patterns (such as the 3-hinge patterns, which are the focus of this work). Moreover, since the initial state of any individual brain is unique to a certain extent, we can expect that tertiary folding patterns are not consistent between any two samples. On the other hand, there are some commonly preserved 3-hinge patterns in all real brains that demonstrate a certain amount of consistency; we thus strive to find an explanation to this consistency.

### Contribution of Fibers on the Location and the Shape of 3-Hinge Patterns

**Figure 8:**
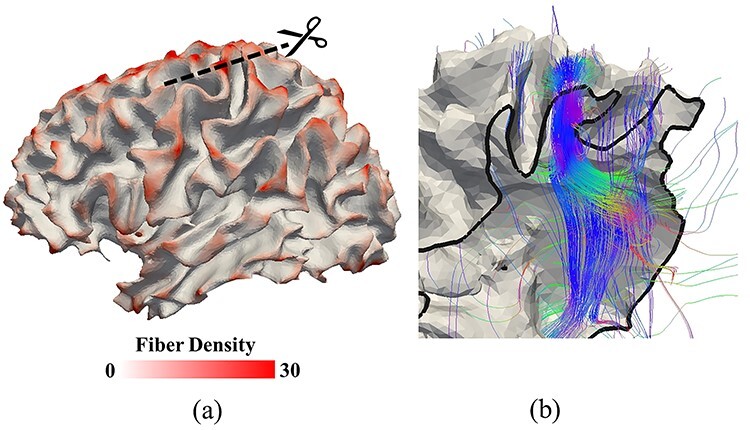
(*a*) Fiber density in the 3-hinge patterns is higher than other locations of a brain (red color regions). The reconstructed surface is white matter surface. (*b*) The cross-section shows how dense fibers are projected to a 3-hinge fold. Black arrow heads in (*a*) and (*b*) highlight the same 3-hinge fold. Diffusion MRI of this subject was used to produce the streamline fibers (color curves in (*b*)) and the white matter surface and the fiber density map on this surface in (*a*). More details are referred to section “Imaging Data Analysis Method”.

In the previous section, we investigated how mechanical parameters are responsible for certain mechanisms of 3-hinge pattern formation. However, those factors alone are not able to determine exact locations and geometry of formed 3-hinge patterns, as shown in Figure 7. Therefore, as discussed previously, there should be other factors that regulate the conserved shape of 3-hinge patterns in a real brain, which have not yet been considered in our FE models. In our previous study (Ge et al. 2018), we showed that, in accompaniment with the differential growth theory, growing fibers could possibly control the location of 3-hinge gyrus formation. Figure 8*a* demonstrates that in the real brain, axonal fibers connected to 3-hinge gyral folds are much denser than those found in other areas.

**Figure 9:**
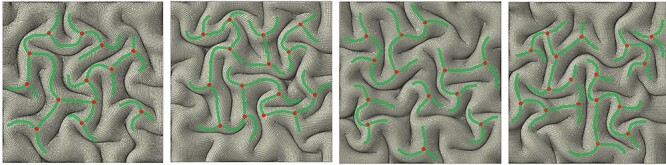
Top view of four FE models with same mechanical and geometrical parameters and different initial perturbations. Locations and 3-hinges patterns are unpredictable.

We obtained average fiber densities of brain locations containing 3-hinge gyral patterns and realized that there is a significant difference in the fiber density between 3-hinge gyral folds and the typical gyral crest lines. Therefore, a possible factor that can be incorporated into FE models to explain certain consistencies in location and shape of 3-hinge patterns is the presence and growth of axonal fibers. To do so, we incorporated the role of axonal wiring into the regulation of location and shape of 3-hinge patterns. We created several models with and without growing fibers with gradient growth rates. The assigning gradient growth to fibers considers that the hinge points have higher growth rate than the hinge lines, because the concentration of fibers around the hinge points is highest, which decreases over the hinge lines with moving away from the hinge points (see Methods section for more details). Figure 9 shows a top view of the final state of four FE models without contribution of fibers. All parameters of models except initial perturbation are the same. The 3-hinge patterns (green lines) and their locations (red dots) are different although their qualitative features are similar to each other, which indicates that the differential growth theory by itself only produces 3-hinge patterns in unpredictable locations. We could not find any specific relationship between the wavelength of the initial perturbation and the location of the formed 3-hinges.

**Figure 10:**
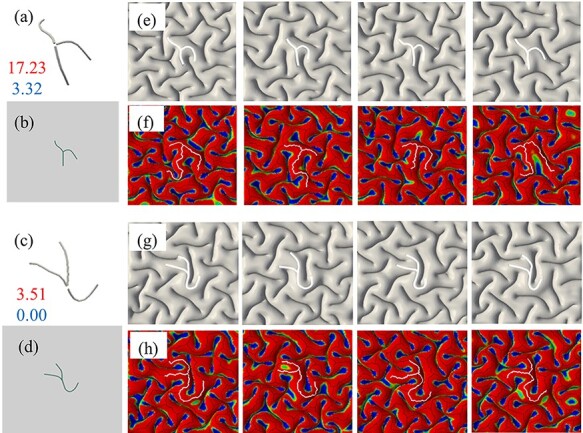
(*a*) and (*c*) Two selected 3-hinge patterns taken from images of a real brain are incorporated into the FE models. (*b*) and (*d*) The top view of FE models in initial state and without growth, with highlighted areas containing fibers. (*e*) and (*g*) Four FE models with growing fibers along the selected 3-hinge patterns shown in (*b*) and (*d*). All parameters of models are same except initial perturbations. After convolution, 3-hinge patterns same as those seen in a real brain form in the center of cortex. (*f*) and (*h*) The 3-hinge patterns (white lines) are detected by the developed algorithm.

As we observed in Figure 7*a*, models without contribution of fibers do not exactly capture 3-hinge patterns and locations same as real images. In the next step, we selected two types of 3-hinge pattern from Figure 7 which were observed in real brain and not in FE models, Figure 10*a*,*c*. We incorporated fibers with gradient growth along the hinge lines following the selected special types of 3-hinge pattern, Figure 10*b*,*d*. These concentrated bundles of fibers project from the base of white matter to the interface of gray–white matters to mimic higher fiber density on 3-hinge patterns. The axial growth rate of the fibers in the hinge points is considered to be the same as the growth rate of the cortex (Ge et al. 2018). This axial growth rate reduces over the hinge lines linearly to zero at the tip of the hinge lines (gradient growth). Figure 10*e*,*g* shows top view of four FE models with growing fibers after growth and convolution. Despite different initial perturbations, we can see that all models formed complex 3-hinge patterns, which have been observed in real brains. Therefore, growing fibers could be a possible factor to determine or regulate the location and shape of 3-hinge patterns. In our previous study, we set the growth rate of axonal fibers as five different values, all of which are comparable with the growth rate in the cortex. Results showed that there is a very high possibility to form a 3-hinge gyrus in the specific area when the growth rate of axonal fibers is close to the growth rate of the cortex, while the area in models without axonal fibers could be located on hinge, sulci, or in-between banks. In addition, the sites with a high density of growing axonal fibers do not develop any sulci in agreement with the experimental results. This study similar to the other studies (Ge et al. 2018; Chavoshnejad et al. 2021) reveals that although fiber bundles do not induce folding, they can regulate the locations of gyri and sulci.

**Figure 11:**
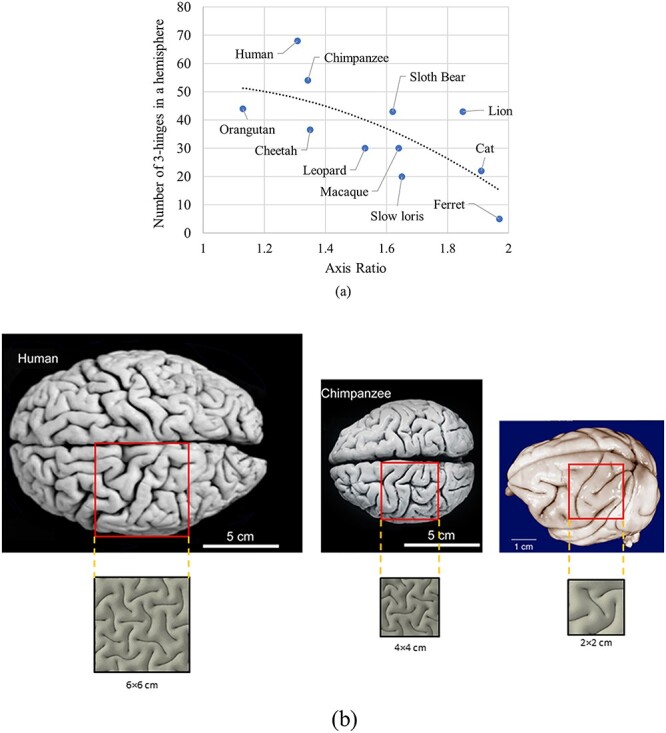
(*a*) Dependence of number of 3-hinge patterns on the axis ratio of brains between different species. The number of 3-hinge patterns is only for a hemisphere. (*b*) Dependence of folding patterns and number of 3-hinge patterns on cortical thickness. In FE models, square patches show representative folding patterns of human, chimpanzee, and macaque brains. The size of the patches is selected to be representative of folding patterns.

**Figure 12:**
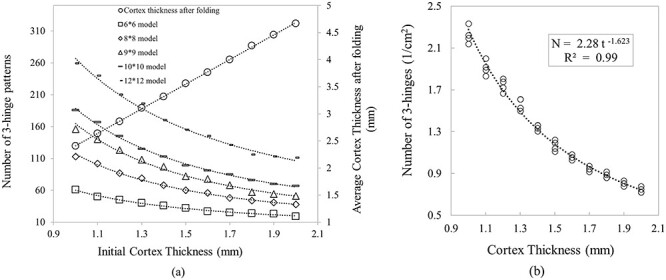
(*a*) Dependency of number of 3-hinge patterns to the surface area and initial thickness of cortex. Numbers on the series legends show the areal dimensions of the cortex in centimeters. (*b*) Dependency of density of 3-hinge patterns in a unit area of models; $N$ is the number of 3-hinge patterns per unit area and $t$ is the initial thickness of cortex.

It is interesting to see that 3-hinge patterns far from their centers are quite different from one another. This result shows that besides the commonly preserved 3-hinge patterns, we can also have various and different 3-hinge patterns in a growing brain such as those illustrated in the previous section. Previous analysis on the relationship between axonal elongation and cortical growth (Holland et al. 2015) concludes that rather than axons pulling on the brain to induce cortical folding, the folding cortex pulls on the axons to trigger axonal elongation and white matter growth. In our models, we assign axial growth to the fibers. With this assumption before instability and folding in the model brain, compressive forces appear in the fibers. But, after the gyrification, this statement is not true and some of the fibers can feel the tension even if they are growing (mostly fibers in the gyri part). This can happen because the differential growth in the gyrification process is a dominating factor to the axonal growth. Therefore, we observe both tensile and compressive fibers at distant locations.

It is worth noting that only the contribution of fibers has been included in the discussion. Admittedly, the axonal wiring is not the only parameter that controls the shape of certain 3-hinges. It is highly possible that axonal wiring together with other factors such as heterogeneity in stiffness and growth and curvature controls the 3-hinges number, location, and shape. For example, the initial curvature of the smooth brain has shown a great impact on the morphology of folds (Budday et al. 2015c; Tallinen et al. 2016). Narrow elongated brains tend to fold mainly in the longitudinal direction, while rounder brains, such as the human brain, fold in the transverse direction (Budday et al. 2015c). Our previous study also showed that the heterogeneous regional growth in the cortex can produce consistent gyrus in a developing brain (Zhang et al. 2016). However, the effect of curvature and heterogeneity in growth and stiffness on the shape and location of 3-hinge patterns in a brain has not yet been thoroughly investigated.

### Effect of the Brain Size and Cortex Thickness on the 3-Hinge Patterns

So far, we have discussed human subjects, but what are the possible patterns of 3-hinges in other primates? DTI data from 64 HCP human brain specimens revealed that the average number of 3-hinge patterns in a human brain is around 137 (Ge et al. 2018), which is considerably greater than the average number of 3-hinge patterns found in chimpanzee and macaque brains, 108 and 60, respectively (Zhang et al. 2020). Brain size could be a possible parameter on the number of 3-hinges, in that bigger brains (with higher prefolding surface area) will have a larger number of 3-hinge patterns. However, results from image data show that different species with similar brain sizes may have a huge difference in the number of 3-hinge patterns (Zhang et al. 2020). Hence, other parameters may control the number of 3-hinge patterns beyond the brain size. Interestingly, Figure 11*a* shows that even species with the same axis ratio of the brain may not have similar numbers of 3-hinge patterns. The axis ratio is the ratio of major axis of the ellipsoidal shape of brain to the minor axis of it (front to rear distance divided by top to bottom distance). As an example, the brains of both a cat and a lion have similar axis ratios but a big difference in the number of 3-hinge patterns. Therefore, neither brain size nor axis ratio is a singular factor in controlling the number of 3-hinge patterns.

Another possible factor that could be considered is the thickness of the cortex. The thickness of the cortex is different between species and is independent from the size or mass of the brain (Sun and Hevner 2014). The average cortical thicknesses for human, chimpanzee, and macaque brains roughly are$2.5,$  $2,$ and$1.68\ \mathrm{mm}$, respectively (Fischl and Dale 2000; Zhang et al. 2017). A previous study analytically indicated that the number of folds increases linearly with the ratio of brain radius to cortex thickness (Budday 2015c). Although the human brain has a higher cortical thickness, it is more convoluted and has a larger number of 3-hinge patterns. This happens because the human brain has larger surface area and the ratio of brain radius to cortex thickness is higher than in both other primates. In our FE models, the average radii for the brains of human, chimpanzee, and macaque were set as$50,32,$ and$26\ \mathrm{mm}$, respectively. Average radius in the patch model is equal to the total height of the model including the thickness of the white and gray matters. Figure 11*b* shows a top view of the FE models for a square patch of every species. The sizes of the patches were selected to be representative of the folding geometry. Figure 11*b* shows that the number of 3-hinge patterns could be a function of cortical thickness. To investigate this relationship, we ran several models for human brains with various cortical thicknesses. The initial cortex thickness varies from$1\ \mathrm{to}\ 2\ \mathrm{mm}$. It is worth mentioning that the cortex thickness after growth and convolution is increased compared with the initial thickness.

Figure 12*a* shows the dependency of the number of 3-hinge patterns and final cortical thickness on the initial cortical thickness and the surface area of the cortex. As can be seen, a cortex with a higher surface area has a higher number of 3-hinge patterns, and a thicker cortex has the trend of forming fewer 3-hinge patterns. Figure 12*a* also shows that cortex thickness after convolution has a linear relationship with the initial thickness of cortex. Since the cortical layer after convolution does not have a uniform thickness, being notably different between sulci and gyri, the thicknesses were roughly calculated and averaged in random locations. To calculate the density of 3-hinge patterns in a unit area for all models presented in Figure 12*a*, a square patch $6\times 6\ \mathrm{cm}$ was cut from the center of models (so as to lessen possible edge effects), and the calculated number of 3-hinge patterns over this area is presented in Figure 12*b*. Based on Figure 12*b* we can, with high accuracy, predict how many 3-hinge patterns will form on the cortex with a known initial cortical thickness and surface area. Furthermore, it is not necessary to have data after convolution; only initial geometrical parameters are required. The human brain in a smooth state, that is, before convolution, has an average surface area of $120\ {\mathrm{cm}}^2$ at 23 weeks of gestation (Zhang et al. 2013) and a cortical thickness of around$1.5\ \mathrm{mm}$. From these numbers, based on Figure 2*b*, we can predict that the human brain after convolution will form roughly “142” 3-hinge patterns, which is comparable with the real brain observed average of 137 (Ge et al. 2018). The discrepancy could possibly be from real brain curvature, which has not been considered in this model. This result shows that by tuning the initial geometrical parameters in a mechanical model brain, we are able to predict the number of such complex 3-hinge patterns after convolution. The result shows that the role of mechanics on the growth and formation of complex shapes in brain is greater than what we previously thought. Future studies are required to help reveal the fundamental contributions of mechanics on the folding of brain and its associated structural disorders.

## Conclusions

Although mechanical forces play a vital role in the evolution of 3-hinge patterns in cortical folding, the fundamentals underlying these active forces that drive this process remain unclear. By using imaging and computational tools, we set out to identify and study the force-generating parameters that have been included in this complex process. We showed that differential growth along with the presence of axonal fibers could be a potential factor in controlling the number, location, and shape of 3-hinge gyral patterns in a developing human brain. The mechanism of the formation of 3-hinge patterns as a part of cortical folding may involve differential growth in a primary role and axonal wiring in a secondary role. From our study, we can speculate that axonal wiring is one of the main contributors to the formation of 3-hinge patterns with certain unique shapes in designated specific locations, as the differential growth hypothesis does not predict the spatially consistent patterns of certain identifiable 3-hinge patterns. It is a challenging and unanswered question of the complex causal relationship: Whether the formation of a 3-hinge pattern then attracts more fibers toward the 3-hinge site, or whether the dense and growing fibers push a specific site to help drive the formation of a 3-hinge pattern initially induced by differential growth. Nevertheless, one clear point is that for the formation of a 3-hinge pattern both differential growth and axonal wiring accompany each other. We also showed that the thickness of the cortex is the main geometrical parameter, which determines the number of 3-hinges patterns in a certain brain surface. However, the size and axis ratio of the brain do not have considerable effect on the number of 3-hinge patterns among different species.

In our work, we choose a small part of the brain with a simple geometry as our basic model to investigate the fundamental of the formation of 3-hinges patterns. This simple model helps us achieve the key findings as we expected; however, future studies with a more realistic model brain will be worthwhile to invest for further exploring the details. The effect of the curvature and heterogeneity in growth and stiffness on the formation of 3-hinge patterns could also be studied. Conducting such a comprehensive model may result in the development of an effective tool to diagnose certain common brain disorders such as autism. Therefore, understanding of 3-hinge gyral patterns formation and development can provide useful insight into the differences between normal and pathological brain function.

## Notes

M.J.R. thanks the start-up support from Department of Mechanical Engineering at Binghamton University. X.W. and T.L. were supported by National Science Foundation (NSF No. 2011369). We would like to thank the Human Connectome Project for sharing the datasets used in this work. *Conflict of Interest:* The authors declare no competing financial interests.

## Ethical Statement

All procedures performed in studies involving human participants were in accordance with the ethical standards of the University of Georgia and with the 1964 Helsinki declaration and its later amendments or comparable ethical standards. All applicable international, national, and/or institutional guidelines for the care and use of animals were followed.
